# Xylazine: A Drug Adulterant of Clinical Concern

**DOI:** 10.1007/s11916-024-01211-z

**Published:** 2024-03-20

**Authors:** Amber N. Edinoff, Saveen Sall, William C. Upshaw, Noah J. Spillers, LeighAnn Y. Vincik, Adalyn S. De Witt, Kevin S. Murnane, Adam M. Kaye, Alan D. Kaye

**Affiliations:** 1 McLean Hospital, Division of Alcohol and Drug Addiction, Belmont, MA 02114 USA; 2 Harvard Medical School, Department of Psychiatry, Boston, MA 02115 USA; 3 Louisiana Addiction Research Center, Louisiana State University Health Shreveport, Shreveport, LA USA; 4 Louisiana State University Health Sciences Center at Shreveport, Department of Psychiatry and Behavioral Medicine, Louisiana State University Health Shreveport, Shreveport, LA USA; 5 Louisiana State University Health Sciences Center at Shreveport, School of Medicine, Shreveport, LA 71103 USA; 6 Louisiana State Health Sciences Center at Shreveport, Department of Anesthesiology, Shreveport, LA 71103 USA; 7 Indiana School of Medicine, Indianapolis, IN 46202 USA; 8 Louisiana State University Health Sciences Center at Shreveport, Department of Pharmacology, Toxicology & Neuroscience, Louisiana State University Health Shreveport, Shreveport, LA USA; 9 Thomas J. Long School of Pharmacy and Health Sciences, University of the Pacific, Department of Pharmacy Practice, Stockton, CA 95211 USA

**Keywords:** Drug adulterants, Xylazine, Fentanyl, Overdose, Skin wounds

## Abstract

**Purpose of Review:**

The opioid epidemic has been responsible for significant morbidity and mortality in the USA and worldwide. As a result, it is essential to recognize the threat these potent drugs can cause when illicitly used. Specifically, introducing fentanyl as a drug adulterant has been shown to impact overdose rates drastically. In this regard, the Drug Enforcement Agency recently released a public safety alert announcing the new threat of a new adulterant called xylazine. Xylazine is a powerful animal sedative with a different mechanism of action when compared to illicit opioids such as heroin and fentanyl. Xylazine is typically injected intravenously via a syringe, often in combination with multiple other drugs. One of the most common drugs, xylazine, is taken in combination with fentanyl, with users of this drug combination describing xylazine as prolonging the euphoric sensation produced by fentanyl.

**Recent Findings:**

Xylazine may cause adverse effects such as bradycardia, brief hypertension followed by hypotension, premature ventricular contractions, ataxia, slurred speech, sedation, and respiratory depression. Much of the recent literature on xylazine use in humans comes from case reports and review articles.

**Summary:**

Related to widespread use in veterinary medicine and increasing circulation in illicit drug markets, there is a critical need for public awareness and additional clinical-based studies to further increase understanding of mediated or modulated pharmacological effects of xylazine in humans. Further research is urgently needed to more clearly understand the implications of unregulated xylazine in the illicit drug market, to formulate public health interventions, and to implement harm reduction strategies.

## Introduction

Drug adulteration is when an illicit drug is contaminated with other substances to increase monetary gain [1]. It is becoming an increasingly concerning topic related to opioids as drug overdose mortality has significantly increased [2]. The opioid epidemic has been responsible for significant morbidity and mortality. Thus, it is vital to appreciate potential threats that these drugs can cause when illicitly used. Specifically, introducing fentanyl as a drug adulterant has been shown to impact overdose drastically. According to the Drug Enforcement Agency (DEA) National Forensic Library Information System data, fentanyl was widely introduced into the illegal drug market starting in 2013, with an exponential increase in fentanyl encounters from drugs confiscated by law enforcement [3]. Since then, overdose numbers have increased from 40,000 to 106,699 deaths reported in 2021 [2]. There was a reported fourfold increase between 1999 and 2008 in overdose deaths [4]. Governmental regulations have aimed to safeguard society from opioid misuse [5]. Recently, the DEA released a public safety alert announcing the new threat of a new adulterant called xylazine [6]. The DEA announced that they had seized xylazine and fentanyl mixtures in 48 of the 50 states. Additionally, the DEA Laboratory System reports that of the fentanyl seized in 2022, 23% of fentanyl powder contained xylazine, while 7% of fentanyl pills were also contaminated with xylazine [6].

Xylazine is a powerful animal sedative with a different mechanism of action when compared to illicit opioids such as heroin and fentanyl. However, there is no reversal agent for xylazine as opposed to the opioid antagonist naloxone for acute opioid overdose treatment. Although there is still no recognized use for xylazine in humans, there has been some discussion regarding the role of xylazine in overdose mortalities. Some arguments include that because xylazine is included as an adulterant, there is less fentanyl, which may protect from overdose death [7]. Contrarily, others say that because both drugs alone contain similar adverse effect profiles, xylazine, as an adulterant with fentanyl, illicit additive, and/or synergistic adverse effects and, therefore, is more responsible for subsequent overdose death [7].

Overdose fatalities involving xylazine have increased by an astounding 1200%. The fatality increase is significant but may be inflated related to a severe lack of uniform screening for the drug by medical examiners. Adverse effects of xylazine in humans can affect breathing, decrease heart rate, and cause severe skin abscesses and ulcers, which may progress to infection, necrosis, and even amputation [7]. Xylazine has also been reported to be addictive, with reports of severe withdrawals, including distress and anxiety. The present investigation, therefore, is a narrative review of drug adulterants, xylazine, and clinical adverse effects.

## History of Drug Adulterants and Overdose

Drug overdose has become an increasingly significant problem in the USA in recent years. In 2020 alone, there were over 80,000 deaths attributed to overdose on one or more drugs. As a result, this represented a 622% increase compared to drug overdose rates in 2000 [8]. The drugs most commonly involved in deaths related to overdose are synthetic opioids, including fentanyl, heroin, and methadone [9]. People often overdose on opioids because of adulterants in the ingested drug [10]. An adulterant in a drug is a pharmacologically active compound that substitutes another drug. The adulterants may be used to increase the effect of another drug, enhance the delivery of a drug, or increase the quantity of drug product produced [11]. The use of these adulterants is a significant issue as many of them are extremely toxic, especially when combined with other illicit drugs [12]. Additionally, adulterants are challenging to test, as many toxicological analyses cannot detect them. Thus, they are not reported when investigating the cause of death in people who die from overdose [11].

Adulterants found in opioids obtained within the United States include acetaminophen, diltiazem, dipyrone, and fentanyl [13]. These adulterants are believed to be linked to health-related issues such as bone marrow damage, cardiac arrhythmia, neutropenia, and renal failure, among other conditions [14]. Fentanyl, in particular, is highly deadly as it is 80–100 times stronger than morphine and thus may be deadly even at low doses [15]. Fentanyl is often used as a substitute for other drugs because it is cheap and inexpensive to manufacture [16]. It may be used knowingly on its own. Many drugs, such as heroin or even nonopioid drugs, such as cocaine or methamphetamines, contain fentanyl as an adulterant, resulting in increased overdose deaths when these drugs are ingested [17, 18].

In recent years, a drug that has been found as an adulterant in other illicit substances, such as fentanyl and cocaine, is xylazine [19]. Xylazine is typically injected intravenously via a syringe, often in combination with multiple other drugs [20]. One of the most common drugs xylazine is taken in combination with fentanyl, with users of this drug combination describing xylazine as prolonging the euphoric sensation produced by fentanyl [21]. The amount of individuals using xylazine has been increasing in recent years, with this rise being most prominent in specific areas such as Puerto Rico, Toronto, and Philadelphia [21]. This increase in the usage of xylazine, especially when taken in combination with opioids such as fentanyl, is troublesome as xylazine has been shown to increase resistance to opioid reversal with naloxone, resulting in an increased amount of deaths from opioid overdose [22]. Thus, it is essential to highlight this trend regarding the rise in the use of xylazine, as the complications that result from taking the drug are potentially fatal.

## Xylazine Overview

Xylazine is used as an analgesic sedative and combined with other general anesthesia drugs in veterinary practice [23]. Xylazine’s mechanism of action is stimulating alpha-2 adrenergic receptors in both the central nervous and peripheral nervous systems and decreasing norepinephrine release in the peripheral nervous system. The central nervous system effects include strong sedation and respiratory depression [23]. Although this is the main understood mechanism of xylazine, there may be additional mechanisms, including dopaminergic, cholinergic, alpha-1 adrenergic, histaminergic, or even opiate mechanisms [23]. With these effects in mind, treating acute toxicity due to xylazine alone primarily focuses on supporting respiratory depression and blood pressure [24]. Subtle fluctuations in thermoregulation can be observed. However, it does not typically require intervention. Intubation and intravenous fluids are typically necessary for stabilizing the patient’s airway to manage changes in respiratory depression and hypotension [23]. Aggressive supportive treatment is often necessary for treating xylazine overdose.

Related to the combination of xylazine, the adverse effects with other drugs known to cause respiratory depression and hypotension result in treating a patient who overdosed on a combination of fentanyl and xylazine challenging. While the opioid antagonist naloxone effectively reverses respiratory depression in opioid overdose, naloxone does not affect respiratory depression caused by xylazine overdose, one of this adulterant’s most concerning characteristics [24]. Another unique characteristic of xylazine as an adulterant is its association with severe dermatological findings, such as severe abscesses, ulcers, and infections [25].

## Pharmacodynamics and Pharmacokinetics

There is consensus that xylazine primarily acts as an agonist at the alpha-2 adrenergic receptor [23]. In this regard, alpha-2 adrenergic receptors are often found on presynaptic axon terminals, which inhibit the release of excess neurotransmitters into the synaptic cleft when stimulated. The typical ligand in the body for alpha-2 adrenergic receptors is norepinephrine, which can diffuse from the synaptic cleft to the receptor located on the membrane of the presynaptic axon terminal. This essentially serves as a negative feedback loop, ensuring that excess neurotransmitter is not released into the synapse [26]. Thus, the stimulation of this receptor by xylazine similarly reduces the release of neurotransmitters such as norepinephrine and serotonin into the synaptic cleft [23]. This has essential physiological consequences as norepinephrine, in particular, plays a critical role in arousal [27]. Evidence for this fact can be seen in how the locus coeruleus, the major site of norepinephrine production in the central nervous system, has a much higher rate of discharge when an individual wakes up than when an individual is asleep [28]. As a result, individuals who inject xylazine tend to feel sedated and relaxed due to the reduced levels of norepinephrine and serotonin in the central nervous system [23]. In addition to being involved in arousal, norepinephrine plays a crucial role in regulating blood pressure. It exerts this effect by binding to alpha-1 receptors on blood vessels with stimulation of these receptors, causing vasoconstriction [29]. Thus, a potential complication caused by xylazine injection is regulating arterial blood pressure [30].

Further research needs to be conducted on the pharmacokinetics of xylazine in humans as there is little data currently on this topic [31]. However, there is a large amount of information regarding the pharmacokinetics of xylazine in various species of animals, such as dogs, sheep, and cattle [32]. In these species of animals, xylazine is distributed very quickly throughout the body and reaches its highest concentration levels in the central nervous system and the kidneys [33]. Xylazine was also shown to have a very high volume of distribution, with values ranging between 1.9 and 2.5 [34]. These high values are believed to be due to xylazine’s lipophilic nature, allowing it to more easily diffuse through the plasma membrane of cells [35]. In addition, the rate of distribution and elimination of xylazine was also very rapid, with the distribution half-life and elimination half-life yielding values between 1.21 and 5.97 min and 23.11 and 49.51 min, respectively. The bioavailability of xylazine had significant variability depending on the animal examined, with the lowest value of 17% reported in a sheep and the highest value of 90% reported in a dog [34]. Peak plasma concentrations of xylazine occurred 12–14 min after drug administration in all animals examined, proving that the drug rapidly affects the body [36]. Regarding the metabolism of xylazine, studies have been conducted in humans examining this process. It has been shown that xylazine may be N-dealkylated, S-dealkylated, oxidized, or hydroxylated based on metabolites detected in human urine samples [37].

## Adverse Effects of Xylazine

Xylazine is only approved for use as an anesthetic in veterinary surgery as it has the potential to cause numerous adverse health effects in humans. People may take xylazine orally, intravenously, intramuscularly, or through inhalation. It may cause adverse effects such as bradycardia, brief hypertension followed by hypotension, premature ventricular contractions, ataxia, slurred speech, sedation, and respiratory depression [25, 33]. The length of how these effects last shows significant variability, with them having the potential to last up to 3 days after the intake of xylazine [25]. Xylazine has also been shown to be extremely toxic to the skin, leading to numerous issues such as cellulitis, skin abscesses, and soft tissue infections [38]. It has been reported that it can cause skin ulcers that are often purulent and may extend all the way to bone [39].

In many cases, these skin injuries may be so severe that amputation of the affected extremity is required [40]. It is believed that issues related to the skin are due to the stimulation of peripheral alpha-2 adrenergic receptors by xylazine, which leads to vasoconstriction of blood vessels in the skin [41]. This vasoconstrictive effect causes a decrease in perfusion to the skin and, thus, reduces its oxygen supply. Further, along with causing adverse skin effects such as those already mentioned, healing of tissue injuries is also impaired due to this lower level of oxygen delivery [38].

In addition to causing the adverse effects mentioned above, xylazine has the potential to be fatal in humans. When taken parenterally, the median lethal dose, or LD_50_, of xylazine is believed to be approximately 15 mg/kg [42]. Xylazine may be especially deadly when it is taken in conjunction with fentanyl, with this combination being responsible for numerous drug overdose deaths [43•]. This may be due to a synergistic effect between the two drugs on depressing respiratory rate [44]. This is especially problematic as naloxone, the typical treatment used in cases of opioid overdose, is ineffective in relieving the physiological effects caused by xylazine intoxication [45]. Instead, treatment for xylazine overdose is typically supportive and involves addressing the physiological effects caused by the drug. Different treatment measures may include endotracheal intubation if respiratory depression is severe, atropine if the heart rate is excessively low, and vasopressor agents if blood pressure falls to dangerous levels [38].

Additionally, several alpha-2 adrenergic receptor antagonists, such as yohimbine and atipamezole, effectively reverse the effects of xylazine in animals. However, these reversal agents have been studied only to a small degree in humans and thus should not be used unless patients are unresponsive to the supportive measures listed above [45]. As patients suffering from an overdose on xylazine often have fentanyl in their system simultaneously, naloxone should still be administered to reverse the adverse effects caused by fentanyl [46]. However, it is crucial for clinicians to recognize that this will not help reverse the effects of xylazine, and thus, other measures must be taken to mitigate its effects [45].

## Xylazine Effects

Much of the recent literature on xylazine use in humans comes from case reports and review articles. Related to widespread use in veterinary medicine and increasing circulation in the illicit drug markets, there is a critical need for additional clinical-based studies to increase awareness of the pharmacological effects of xylazine in humans. Expanding data collection and establishing a consensus on human xylazine use would benefit the general population and clinicians since there is no definitive treatment protocol for intoxication or withdrawal. Although xylazine’s association with adverse outcomes is evident in the multiple case reports reviewed, motivations of those who use/supply, national trends, geographic distribution, and long-term health risks are poorly characterized. Further research is urgently needed to fully understand the implications of unregulated xylazine in the illicit drug market, formulate public health interventions, and implement harm reduction strategies.

## Demographics and Trends

Xylazine has been a known adulterant of illicit opioids in Puerto Rico since the early 2000s but has more recently been documented in urban Philadelphia. Present in almost one-third of opioid overdose deaths reported in 2019, Philadelphia overwhelmingly appears to be the earliest and most significant known area of xylazine use in the USA [47]. The Philadelphia Public Health Department conducted a 10-year study to describe trends and characteristics of unintentional deaths from heroin and/or fentanyl overdose containing xylazine in Philadelphia. Results showed that xylazine went from being detected in less than 2% of cases of fatal heroin and/or fentanyl overdose between 2010 and 2015 to 31% of the 858 fatal heroin and/or fentanyl overdose cases in 2019 [47]. Evidence of this increase is evident across multiple studies, including a 2021 study conducted at Thomas Jefferson University Hospital in Philadelphia. Korn et al. developed an LC–MS/MS assay and identified 81 inpatients who had screened positive for fentanyl; of these xylazine was identified in 63 [48•].

The rising trend documented in Philadelphia is being mirrored on a smaller scale nationwide. Using data from the Center for Disease Control's State Unintentional Drug Overdose Reporting System, Karissa et al. noted that among 21 jurisdictions, which included 20 states and the District of Columbia, the monthly percentage of illicitly manufactured fentanyl involved deaths with xylazine detected increased from 2.9% in January 2019 to 10.9% in June 2022, an increase of 276%. Interestingly, this study also concluded that no particular demographic group was disproportionately affected by xylazine. However, the adulterant was detected in a higher percentage of jurisdictions in the Northeast and lower in the West US Census Bureau region [49].

Supporting this Northeast predominance and trend to spread westward, a study by Friedman et al. systematically searched all four US Census Regions for records describing xylazine-present overdose and reported that the prevalence of xylazine rose 1761% in five years. Results showed an increase from 0.36% of overdose deaths in 2015 to 6.7% in 2020 [21]. The highest xylazine prevalence data was observed in Philadelphia with 25.8% of deaths, followed by Maryland and Connecticut with a pattern similar to the trajectory of illicitly manufactured fentanyl in recent years [21]. This is believed to be no incidental finding since fentanyl was found in 98% of xylazine-related deaths [21]. A large analytical study in West Virginia also showed that 98% of xylazine decedents had co-intoxications with fentanyl and the second most common methamphetamines. Sibbesen et al. noted that 117 of the 3292 drug deaths involved xylazine, increasing yearly from 1% in 2019 to 5% in mid-2021. Furthermore, a significantly greater history of drug or alcohol misuse and hepatic disease was noted in xylazine decedents [50].

As a result, overdose rates doubled in Cook County, IL from 2019 to 2022 [51]. The researchers attempted to localized geographic clustering of xylazine-involved fatal overdoses in Cook County, IL, to better understand shared geographic and forensic features of other substances such as fentanyl, alcohol, and stimulants. The study found that 94.4% of xylazine-positive overdoses contained fentanyl, supporting their conclusion that xylazine fatal overdose incident locations exhibited localized clustering relative to fentanyl overdoses [51]. However, the study was unable to locate specific high-risk clustering of xylazine but presented evidence that they occurred closer in proximity of up to 16 miles compared to fentanyl overdoses. Further research is essential to provide insight into how drug-related outcomes are distributed locally. This understanding will help identify locations suitable for potential interventions and public engagement and predict warning signs at the community level.

## Mixed Perspectives on the Effects of Xylazine in Case Reports

As xylazine prevalence continues to increase nationwide, many authors question why it is used as an adulterant for illicit drugs. Research about the perspectives of people who inject drugs on the effects of xylazine use is mixed. Participants in a recent study were interviewed in Philadelphia between January and May 2021, and xylazine in the fentanyl and heroin supply was discussed. The 13 participants had used fentanyl or heroin, and the unanimous consensus of this study showed that no participant enjoyed the addition of xylazine or sought to have it in their fentanyl or heroin. They reported dissatisfaction with the sensation of the drug and had many safety concerns about xylazine exposure, including tranquilizer dependence [52]. Participants of this study expressed a desire for the production and distribution of xylazine test strips to limit unwanted exposures.

On the contrary, another study transcribed interviews from people who inject drugs in Philadelphia, PA, in which participants were not as unanimously opposed but spoke with a mix of intrigue and apprehension about the psychoactive effects of xylazine. It was stated by interviewees that opioid formulations containing xylazine are highly sought-after and that the addition of xylazine heightens the euphoria and prolongs the duration of fentanyl injections [21]. Participants noted individuals known to sell xylazine-containing products on the illicit drug market to have more business. Despite this, the interviewees acknowledged the risks associated with the adulterant, particularly concerns about over-sedation and increased risk of soft tissue damage. One interviewee stated that if xylazine is not injected properly, it can cause the whole upper extremity to turn black. It is suspected that once participants develop a skin ulcer, they may continually inject at the ulcer site to alleviate the pain [47]. An interviewee also recounted her negative experience of the drug, causing her to black out, have amnesia, and participate in activities unknowingly, such as walking out in front of a car. Another stated that while using xylazine, she felt like a zombie, with slowed, un-purposeful movements, ultimately causing her to fall and sustain a fracture to her skull.

Interestingly, interviewees noted that formulations of drugs that included xylazine could be distinguished from others immediately upon injection because of a dry mouth and a characteristic taste. It seems both the adverse health effects and potential higher potency euphoric effects are well known to consumers, and the consumer decides the risk and preference. Further qualitative studies based on the perspectives of people who inject drugs are needed to understand better the motivations and circumstances leading to xylazine use and why xylazine might be spreading across the USA.

## Management of Xylazine Misuse and Withdrawal

It has been established that the clinical picture of xylazine intoxication mimics that of an opioid. Limited studies suggest presentation in humans include central nervous system depression, hypotension, bradycardia, drowsiness, lethargy, and possible withdrawal symptoms with chronic use. The literature cautions that these similar pharmacologic effects may create synergistic toxic effects in humans, so health professionals should be aware of possible enhanced toxicity from xylazine ingestion, especially since naloxone is not a proven antidote to xylazine’s adverse effects.

Love et al. evaluated emergency department patients between 2020 and 2021 to investigate clinical outcome differences for patients with and without xylazine exposures. The study interestingly concluded that patients testing positive for xylazine with illicit opioid overdose showed less severe outcomes, with lower rates of cardiac arrest and coma [53]. A systematic review by Ayub et al. assessed the impact of xylazine use in 59 cases and found that intravenous administration was the most common route, with overall doses ranging from 40 to 4300 mg. Of the 59 cases studied, 21 resulted in fatal outcomes, 17 involving xylazine use in conjunction with other drugs. The average dose in fatal cases was 1200 mg, compared to 525 mg in non-fatal cases [54]. Currently, there are no FDA-approved medications for treating xylazine intoxication or managing xylazine withdrawal, and there is little testing capable of identifying xylazine in drug supplies, limiting the development of novel treatment strategies.

A case series of accidental xylazine intoxication in two individuals who worked with livestock discussed treatment and challenged the efficacy of naloxone usage in xylazine intoxication. The first patient presented in a reduced conscious state, normotensive but bradycardic less than an hour after the unintentional injection of the tranquilizer in his left arm. Supplemental oxygen and IV atropine 0.5 mg were given, and his heart rate improved slightly. Due to the patient’s continued central nervous system depression, poison control recommended naloxone. IV naloxone was administered, but the patient did not show significant improvement in consciousness. Eventually, he regained full consciousness after 24 h and was safely discharged after 48 h. In the second case of the series, the patient presented to the emergency department alert and could give his history but soon became drowsy only minutes after arrival. He was hypotensive and bradycardic, but in contrast to the previous case, this patient was not treated with naloxone. He was given supportive treatment with oxygen, fluids, atropine, and noradrenaline and fully recovered within 24 h [55•]. Similar outcomes were observed in both cases; therefore, the role of naloxone in xylazine toxicity in humans remains uncertain. The case series concluded that general management of xylazine intoxication should focus on supportive care, including ventilation and hemodynamics. In the case of an accidental injection that can occur in individuals who work in close proximity to livestock or veterinary activities, it raises an essential point of management for ER physicians.

A single case study discussed a potentially safe and comfortable medication for xylazine withdrawal in hospitalized patients. The patient was a chronically used xylazine and was experiencing symptoms of withdrawal and lower extremity wounds believed to be caused by xylazine injection. She was treated with an infusion of the sedative dexmedetomidine in combination with phenobarbital and tizanidine. Tizanidine was later transitioned to clonidine. After four days on this regimen, the patient was no longer experiencing withdrawal symptoms, suggesting that xylazine withdrawal can be pharmacologically managed in an inpatient setting [56]. Table 1 is a summary of the clinical information discussed in this section.

**Table 1:** Summary of xylazine clinical studies

**Author, year published, and citation**	**Study title and description**	**Results**	**Conclusions**
Delcher et al. (2023) [51]	*Xylazine-involved fatal overdoses and localized geographic clustering: Cook County, IL, 2019–2022* This study conducted a geographic analysis to better identify the spatial distribution of xylazine-involved fatal overdoses compared to other substances in Cook County, IL	Analysis showed that in 2019–2022 in Cook County, IL, 94.4% of xylazine-positive overdoses contained fentanyl. The study was unable to locate high-risk clustering of xylazine-involved fatal overdoses but presented evidence that they occurred closer in proximity of up to 16 miles compared to fentanyl overdoses	Xylazine fatal overdose incident locations exhibited localized clustering primarily relative to fentanyl overdoses, but clusters were not precisely detected at these scales
Sibbesen et al. (2023) [50]	*Characteristics of xylazine-related deaths in West Virginia-Xylazine-related deaths* This study conducted a large analysis comparing the toxicological findings between xylazine and nonxylazine deaths between 2019 to mid-2021 in West Virginia	Analysis showed 117 of the 3292 drug deaths involved xylazine, increasing from 1% in 2019 to 5% in mid-2021. Xylazine decedents primarily had co-intoxication with fentanyl, followed by methamphetamines. A significantly greater history of drug or alcohol misuse and hepatic disease was noted in xylazine decedents	Identification of xylazine is steadily increasing in West Virginia, especially co-intoxication with fentanyl
Karissa et al. (2023) [49]	*Illicitly Manufactured Fentanyl-Involved Overdose Deaths with Detected Xylazine—United States, January 2019-June 2022* This report uses data from the CDC’s State Unintentional Drug Overdose Reporting System (SUDORS) to describe illicitly manufactured fentanyl (IMF) involved overdose deaths with and without xylazine detected that occurred during January 2019-June 2022	Among 20 states and the District of Columbia, the monthly percentage of IMF-involved deaths with xylazine detected increased from 2.9 to 10.9%. Xylazine was detected in a higher percentage of jurisdictions in the Northeast and lower in the West US Census Bureau regions. Finally, demographic characteristics, drug co-involvement, and circumstances were similar among IMF-involved deaths with and without xylazine detected	No demographic groups are disproportionately affected by xylazine, and certain drugs are not more often used with IMF products versus without xylazine
Korn et al. (2021) [48•]	*High prevalence of xylazine among fentanyl screen-positive urines from hospitalized patients, Philadelphia, 2021* This study created an LC–MS/MS assay to determine the prevalence of xylazine among fentanyl screen-positive urine samples at Thomas Jefferson University Hospital	Among 81 urine samples screened positive for fentanyl, 78% were positive for xylazine	It was concluded by LC–MS/MS that there was a high prevalence of xylazine in fentanyl screen-positive urine samples submitted to the hospital laboratory
Ehrman-Dupre et al. (2022) [56]	*Management of Xylazine Withdrawal in a Hospitalized Patient: A Case Report* This case illustrates a potential treatment pathway for xylazine withdrawal in hospitalized patients	The patient was treated with an infusion of the sedative dexmedetomidine in combination with phenobarbital and tizanidine. Her muscle relaxant was later transitioned to clonidine	After four days on this regimen, the patient was no longer experiencing withdrawal symptoms, suggesting that xylazine withdrawal can be pharmacologically managed in an inpatient setting
Friedman et al. (2022) [21]	*Xylazine spreads across the US: A growing component of the increasingly synthetic and polysubstance overdose crisis* This study systematically searched for records describing xylazine-present overdose mortality across the US and assessed time trends and overlap with other drugs. They also gathered ethnographic data from people who inject drugs (PWID), investigating their perspective on increasing xylazine prevalence	Among 10 jurisdictions representing all US census regions, the prevalence of xylazine rose from 0.36% of overdose deaths in 2015 to 6.7% in 2020, with the highest deaths observed in Philadelphia. Fentanyl was found in 98% of xylazine-related deaths. PWID stated that xylazine improved the euphoria and prolonged the duration of fentanyl injections, but often left the user over-sedated or with soft tissue wounds	Xylazine is increasingly present in all US census regions in overdose deaths and is significantly linked to illicitly manufactured fentanyl Ethnographic data suggests that xylazine is sought due to its heightened psychoactive effects
Love et al. (2023) [53]	*Opioid overdoses involving xylazine in emergency department patients: a multicenter study* This study evaluated ED patients between 2020 and 2021 for clinical outcome differences for patients with and without xylazine exposures	321 patients were tested, 90 of whom were positive for xylazine. Analysis of positive patient outcomes showed odds of cardiac arrest adjusted OR 0.30, 95% CI 0.10–0.92 and coma adjusted OR 0.52, 95% CI 0.29–0.94	Patients testing positive for xylazine with illicit opioid overdose showed cardiac arrest, and coma outcomes occurred significantly less
Choon et al. (2023) [55•]	*A case series of accidental xylazine intoxication in humans: Is there a role of naloxone as an antidote?* This case report discusses two accidental human injections with xylazine and challenges the efficacy and benefit of naloxone usage in xylazine intoxication	The first patient was treated with oxygen, IV atropine, and IV naloxone and fully recovered within 24 h The second patient was given supportive treatment with oxygen, fluids, atropine, and noradrenaline and fully recovered within 24 h	Similar outcomes were observed in both cases, so the role of naloxone in xylazine toxicity in humans remains uncertain. General management of xylazine intoxication should focus on supportive care including ventilation and hemodynamics
Johnson et al. (2021) [47]	*Increasing presence of xylazine in heroin and/or fentanyl deaths, Philadelphia, Pennsylvania, 2010–2019* This 10-year study describes trends and characteristics of unintentional deaths from heroin and/or fentanyl overdose with xylazine detections occurring in Philadelphia, Pennsylvania	Between 2010 and 2015, xylazine was detected in 2% of unintentional overdose deaths with heroin and/or fentanyl detections, increasing to 11% in 2016, 10% in 2017, 18% in 2018, and 31% in 2019	Xylazine detections are evident and steadily increasing in Philadelphia, Pennsylvania
Ayub et al. (2023) [54]	*Xylazine in the Opioid Epidemic: A Systematic Review of Case Reports and Clinical Implications* This systematic review assessed the impact of xylazine use and overdoses within the context of the opioid epidemic	Intravenous (IV) administration was a common route for xylazine use, with doses ranging from 40 to 4300 mg. The average dose in fatal cases was 1200 mg, compared to 525 mg in non-fatal cases	This paper summarized the complex nature of xylazine use and the urgent need for a comprehensive approach to its detection, management, and harm reduction
Reed et al. (2023) [52]	*Perspective of people in Philadelphia who use fentanyl/heroin adulterated with the animal tranquilizer xylazine; Making a case for xylazine test strips* This study transcribed and analyzed interviews with PWID in which participants were questioned about xylazine and hypothetical xylazine test strips	All 13 participants unanimously disliked the addition of xylazine to their drug supply and had safety concerns about xylazine exposure. All were interested in hypothetical xylazine test strips	In the present study, people who use fentanyl/heroin indicated an interest in testing their drug for the presence of xylazine prior to use

## Conclusions

Drug adulterants are becoming an increasingly concerning topic regarding public health. The history of drug adulterants illustrates the vast detrimental impact it can have on overdose mortality. Xylazine has been shown to complicate the treatment of opioid overdose. The confounding adverse effects of both illicit opioids and xylazine, in addition to no reversal agent for xylazine, prove to be a challenge when addressing overdose mortalities. Due to little data on xylazine's effects on humans, there is still a certain degree of ambiguity regarding the complete mechanism of xylazine. The challenge of recognizing an overdose due to a drug being cut with xylazine further hinders potential lifesaving treatment. The vasoconstrictive actions of adrenergic receptors seem to cause prolonged wounds, which may prevent the ability for further treatment and, as a result, progress to necrotic wounds. There is still some discussion about whether xylazine increases mortality through acute overdose or subsequent necrotic wounds and infections. Regardless, it is commonly accepted that xylazine has no use in humans. More studies are needed to analyze pharmacological characteristics to identify a treatment for acute xylazine overdose. In addition to these physiologic factors, the fact that xylazine is not a controlled substance further complicates the issue as there is no punishment for distributing it.

## Data Availability

All data is publically available on the cited references in pubmed.

## References

[1] Schiller EY, Goyal A, Mechanic OJ. Opioid overdose. In: StatPearls [Internet]. Treasure Island (FL): StatPearls Publishing; 2023 [cited 2023 Aug 6]. Available from: https://www.ncbi.nlm.nih.gov/books/NBK470415/.

[2] National Institute on Drug Abuse [Internet]. 2023 [cited 2023 Aug 6]. Drug overdose death rates. Available from: https://nida.nih.gov/research-topics/trends-statistics/overdose-death-rates.

[3] Reported law enforcement encounters testing positive for fentanyl increase across US | Drug overdose | CDC Injury Center [Internet]. 2023 [cited 2023 Aug 6]. Available from: https://www.cdc.gov/drugoverdose/deaths/fentanyl-encounters/index.html.

[4] Jones MR, Viswanath O, Peck J, Kaye AD, Gill JS, Simopoulos TT (2018). A brief history of the opioid epidemic and strategies for pain medicine. Pain Ther.

[5] Jones MR, Novitch MB, Sarrafpour S, Ehrhardt KP, Scott BB, Orhurhu V (2019). Government legislation in response to the opioid epidemic. Curr Pain Headache Rep.

[6] DEA reports widespread threat of fentanyl mixed with xylazine | DEA.gov [Internet]. [cited 2023 Aug 6]. Available from: https://www.dea.gov/alert/dea-reports-widespread-threat-fentanyl-mixed-xylazine.

[7] AP News [Internet]. 2023 [cited 2023 Aug 6]. Animal sedative xylazine in fentanyl is causing wounds and scrambling efforts to stop overdoses. Available from: https://apnews.com/article/xylazine-tranquilizer-animal-sedative-opioids-overdose-eabdd7332f309e9e9b610db0c2c9922c.

[8] Underlying cause of death, 1999–2020 Request [Internet]. [cited 2023 Aug 5]. Available from: https://wonder.cdc.gov/ucd-icd10.html.

[9] National Institute on Drug Abuse [Internet]. 2021 [cited 2023 Aug 5]. Fentanyl drugfacts. Available from: https://nida.nih.gov/publications/drugfacts/fentanyl.

[10] Centers for Disease Control and Prevention [Internet]. 2023 [cited 2023 Aug 5]. Other drugs. Available from: https://www.cdc.gov/drugoverdose/deaths/other-drugs.html.

[11] Cole C, Jones L, McVeigh J, Kicman A, Syed Q, Bellis M (2011). Adulterants in illicit drugs: a review of empirical evidence. Drug Test Anal.

[12] Singh VM, Browne T, Montgomery J (2019). The emerging role of toxic adulterants in street drugs in the US illicit opioid crisis. Public Health Rep.

[13] Fiorentin TR, Krotulski AJ, Martin DM, Browne T, Triplett J, Conti T (2019). Detection of cutting agents in drug-positive seized exhibits within the United States. J Forensic Sci.

[14] Phillips KA, Hirsch GA, Epstein DH, Preston KL (2012). Cardiac complications of unwitting co-injection of quinine/quinidine with heroin in an intravenous drug user. J Gen Intern Med.

[15] Ramos-Matos CF, Bistas KG, Lopez-Ojeda W. Fentanyl. In: StatPearls [Internet]. Treasure Island (FL): StatPearls Publishing; 2023 [cited 2023 Aug 5]. Available from: https://www.ncbi.nlm.nih.gov/books/NBK459275/. 29083586

[16] Fentanyl’s rise on darknet markets (and how to stop it) [Internet]. Chicago Policy Review. 2020 [cited 2023 Aug 5]. Available from: https://chicagopolicyreview.org/2020/08/10/fentanyls-rise-on-darknet-markets-and-how-to-stop-it/.

[17] Scholl L. Drug and opioid-involved overdose deaths — United States, 2013–2017. MMWR Morb Mortal Wkly Rep [Internet]. 2019 [cited 2023 Aug 5];67. Available from: https://www.cdc.gov/mmwr/volumes/67/wr/mm675152e1.htm. 10.15585/mmwr.mm675152e1 PMC6334822 30605448

[18] McKnight C, Des Jarlais DC (2018). Being “hooked up” during a sharp increase in the availability of illicitly manufactured fentanyl: adaptations of drug using practices among people who use drugs (PWUD) in New York City. Int J Drug Policy.

[19] Krishnan M. A horrifying drug called ‘tranq dope’ is spreading in the US [Internet]. Vice. 2022 [cited 2023 Aug 5]. Available from: https://www.vice.com/en/article/akeqje/tranq-dope-in-united-states.

[20] Reyes JC, Negrón JL, Colón HM, Padilla AM, Millán MY, Matos TD (2012). The emerging of xylazine as a new drug of abuse and its health consequences among drug users in Puerto Rico. J Urban Health Bull N Y Acad Med.

[21] Friedman J, Montero F, Bourgois P, Wahbi R, Dye D, Goodman-Meza D (2022). Xylazine spreads across the US: a growing component of the increasingly synthetic and polysubstance overdose crisis. Drug Alcohol Depend.

[22] Khatri SN, Sadek S, Kendrick PT, Bondy EO, Hong M, Pauss S, et al. Xylazine suppresses fentanyl consumption during self-administration and induces a unique sex-specific withdrawal syndrome that is not altered by naloxone in rats. Exp Clin Psychopharmacol. 2023. 10.1037/pha0000670 PMC10799160 37470999

[23] Hoffmann U, Meister CM, Golle K, Zschiesche M (2001). Severe intoxication with the veterinary tranquilizer xylazine in humans. J Anal Toxicol.

[24] National Institute on Drug Abuse [Internet]. 2022 [cited 2023 Aug 6]. Xylazine. Available from: https://nida.nih.gov/research-topics/xylazine.

[25] Ruiz-Colón K, Chavez-Arias C, Díaz-Alcalá JE, Martínez MA (2014). Xylazine intoxication in humans and its importance as an emerging adulterant in abused drugs: a comprehensive review of the literature. Forensic Sci Int.

[26] Philipp M, Brede M, Hein L (2002). Physiological significance of α2-adrenergic receptor subtype diversity: one receptor is not enough. Am J Physiol-Regul Integr Comp Physiol.

[27] Foote SL, Bloom FE, Aston-Jones G (1983). Nucleus locus ceruleus: new evidence of anatomical and physiological specificity. Physiol Rev.

[28] Foote SL, Aston-Jones G, Bloom FE (1980). Impulse activity of locus coeruleus neurons in awake rats and monkeys is a function of sensory stimulation and arousal. Proc Natl Acad Sci U S A.

[29] Taylor BN, Cassagnol M. Alpha-adrenergic receptors. In: StatPearls [Internet]. Treasure Island (FL): StatPearls Publishing; 2023 [cited 2023 Aug 6]. Available from: https://www.ncbi.nlm.nih.gov/books/NBK539830/. 30969652

[30] Hsu WH, Lu ZX, Hembrough FB (1985). Effect of xylazine on heart rate and arterial blood pressure in conscious dogs, as influenced by atropine, 4-aminopyridine, doxapram, and yohimbine. J Am Vet Med Assoc.

[31] Gupta R, Holtgrave DR, Ashburn MA (2023). Xylazine — medical and public health imperatives. N Engl J Med.

[32] Garcia-Villar R, Toutain PL, Alvinerie M, Ruckebusch Y (1981). The pharmacokinetics of xylazine hydrochloride: an interspecific study. J Vet Pharmacol Ther.

[33] Velez LI, Shepherd G, Mills LD, Rivera W (2006). Systemic toxicity after an ocular exposure to xylazine hydrochloride. J Emerg Med.

[34] The pharmacokinetics of xylazine hydrochloride: an interspecific study - GARCIA‐VILLAR - 1981 - Journal of Veterinary Pharmacology and Therapeutics - Wiley Online Library [Internet]. [cited 2023 Aug 6]. Available from: https://onlinelibrary.wiley.com/doi/10.1111/j.1365-2885.1981.tb00715.x. 10.1111/j.1365-2885.1981.tb00715.x 7349331

[35] Mansoor A, Mahabadi N. Volume of distribution. In: StatPearls [Internet]. Treasure Island (FL): StatPearls Publishing; 2023 [cited 2023 Aug 6]. Available from: https://www.ncbi.nlm.nih.gov/books/NBK545280/.

[36] Carruthers SG, Nelson M, Wexler HR, Stiller CR (1979). Xylazine hydrochloridine (Rompun) overdose in man. Clin Toxicol.

[37] Meyer GMJ, Maurer HH (2013). Qualitative metabolism assessment and toxicological detection of xylazine, a veterinary tranquilizer and drug of abuse, in rat and human urine using GC–MS, LC–MSn, and LC–HR-MSn. Anal Bioanal Chem.

[38] Liu CM, Chiu MJ, Fang CC, Chen WJ (2007). Letter to the editor: “Xylazine abuse: a rare cause of syncope”. Clin Toxicol.

[39] Malayala SV, Papudesi BN, Bobb R, Wimbush A. Xylazine-induced skin ulcers in a person who injects drugs in Philadelphia, Pennsylvania, USA. Cureus [Internet]. 2022 Aug 19 [cited 2023 Aug 6]; Available from: https://www.cureus.com/articles/98408-xylazine-induced-skin-ulcers-in-a-person-who-injects-drugs-in-philadelphia-pennsylvania-usa. 10.7759/cureus.28160 PMC9482722 36148197

[40] Soderquist M, Delgado G, Abdelfattah H, Thoder J, Solarz M. Necrotic upper-extremity infections in people who inject drugs: a case series. J Hand Surg [Internet]. 2023 May 12 [cited 2023 Aug 6];0(0). Available from: https://www.jhandsurg.org/article/S0363-5023(23)00177-6/fulltext. 10.1016/j.jhsa.2023.04.001 37178065

[41] Link RE, Desai K, Hein L, Stevens ME, Chruscinski A, Bernstein D (1996). Cardiovascular regulation in mice lacking alpha2-adrenergic receptor subtypes b and c. Science.

[42] Gallanosa AG, Spyker DA, Shipe JR, Morris DL (1981). Human xylazine overdose: a comparative review with clonidine, phenothiazines, and tricyclic antidepressants. Clin Toxicol.

[43] Thangada S, Clinton HA, Ali S, Nunez J, Gill JR, Lawlor RF (2021). Notes from the field: xylazine, a veterinary tranquilizer, identified as an emerging novel substance in drug overdose deaths — Connecticut, 2019–2020. Morb Mortal Wkly Rep.

[44] Busardò FP, Pichini S, Pacifici R, Karch SB (2016). The never-ending public health issue of adulterants in abused drugs. J Anal Toxicol.

[45] Capraro AJ, Wiley JF, Tucker JR (2001). Severe intoxication from xylazine inhalation. Pediatr Emerg Care.

[46] Jordan MR, Morrisonponce D. Naloxone. In: StatPearls [Internet]. Treasure Island (FL): StatPearls Publishing; 2023 [cited 2023 Aug 6]. Available from: https://www.ncbi.nlm.nih.gov/books/NBK441910/.

[47] Johnson J, Pizzicato L, Johnson C, Viner K. Increasing presence of xylazine in heroin and/or fentanyl deaths, Philadelphia, Pennsylvania, 2010–2019 | Injury Prevention [Internet]. [cited 2023 Sep 28]. Available from: https://injuryprevention.bmj.com/content/27/4/395.long. 10.1136/injuryprev-2020-043968 33536231

[48] Korn WR, Stone MD, Haviland KL, Toohey JM, Stickle DF (2021). High prevalence of xylazine among fentanyl screen-positive urines from hospitalized patients, Philadelphia, 2021. Clin Chim Acta Int J Clin Chem.

[49] Kariisa M, O’Donnell J, Kumar S, Mattson CL, Goldberger BA (2023). Illicitly manufactured fentanyl–involved overdose deaths with detected xylazine — United States, January 2019–June 2022. Morb Mortal Wkly Rep.

[50] Sibbesen J, Abate MA, Dai Z, Smith GS, Lundstrom E, Kraner JC (2023). Characteristics of xylazine-related deaths in West Virginia—Xylazine-related deaths. Am J Addict.

[51] Delcher C, Anthony N, Mir M (2023). Xylazine-involved fatal overdoses and localized geographic clustering: Cook County, IL, 2019–2022. Drug Alcohol Depend.

[52] Reed MK, Imperato NS, Bowles JM, Salcedo VJ, Guth A, Rising KL (2022). Perspectives of people in Philadelphia who use fentanyl/heroin adulterated with the animal tranquilizer xylazine; making a case for xylazine test strips. Drug Alcohol Depend Rep.

[53] Love JS, Levine M, Aldy K, Brent J, Krotulski AJ, Logan BK (2023). Opioid overdoses involving xylazine in emergency department patients: a multicenter study. Clin Toxicol Phila Pa.

[54] Ayub S, Parnia S, Poddar K, Bachu AK, Sullivan A, Khan AM, et al. Xylazine in the opioid epidemic: a systematic review of case reports and clinical implications. Cureus. 2023;15(3):e36864. 10.7759/cureus.36864 PMC10063250 37009344

[55] Choon LK, Khiruddin AI, Annuar WMWM, Shamsuddin SR (2023). A case series of accidental xylazine intoxication in humans; is there a role of naloxone as an antidote?. Turk J Emerg Med.

[56] Ehrman-Dupre R, Kaigh C, Salzman M, Haroz R, Peterson LK, Schmidt R (2022). Management of xylazine withdrawal in a hospitalized patient: a case report. J Addict Med.

